# Hypoxia Exerts Dualistic Effects on Inflammatory and Proliferative Responses of Healthy and Asthmatic Primary Human Bronchial Smooth Muscle Cells

**DOI:** 10.1371/journal.pone.0089875

**Published:** 2014-02-24

**Authors:** Laura Keglowich, Melissa Baraket, Michael Tamm, Peter Borger

**Affiliations:** 1 Pulmonary Cell Research, Department of Biomedicine, University of Basel, Basel, Switzerland; 2 Sydney Medical School, The University of Sydney, Sydney, Australia; 3 Department of Pneumology, University Hospital Basel, Basel, Switzerland; University of Giessen Lung Center, Germany

## Abstract

**Background:**

For oxygen supply, airway wall cells depend on diffusion though the basement membrane, as well as on delivery by micro-vessels. In the asthmatic lung, local hypoxic conditions may occur due to increased thickness and altered composition of the basement membrane, as well as due to edema of the inflamed airway wall.

**Objective:**

In our study we investigated the effect of hypoxia on proliferation and pro-inflammatory and pro-angiogenic parameter production by human bronchial smooth muscle cells (BSMC). Furthermore, conditioned media of hypoxia-exposed BSMC was tested for its ability to induce sprout outgrowth from endothelial cells spheroids.

**Methods:**

BSMC were cultured in RPMI1640 (5% FCS) under normoxic (21% O_2_) and hypoxic (1% and 5% O_2_) conditions. Proliferation was determined by cell count and Western blot analysis for cyclin E and Proliferating Cell Nuclear Antigen (PCNA). Secretion of IL-6, IL-8, ENA-78 and VEGF-A was analyzed by ELISA. BSMC conditioned medium was tested for its angiogenic capacity by endothelial cell (EC)-spheroid *in vitro* angiogenesis assay.

**Results:**

Proliferation of BSMC obtained from asthmatic and non-asthmatic patients was significantly reduced in the presence of 1% O2, whereas 5% O2 reduced proliferation of asthmatic BSMC only. Hypoxia induced HIF-1α expression in asthmatic and non-asthmatic BSMC, which coincided with significantly increased release of IL-6, IL-8 and VEGF-A, but not ENA-78. Finally, endothelial sprout outgrowth from EC spheroids was increased when exposed to hypoxia conditioned BSMC medium.

**Conclusion:**

Hypoxia had dualistic effects on proliferative and inflammatory responses of asthmatic and non-asthmatic BSMC. First, hypoxia reduced BSMC proliferation. Second, hypoxia induced a pro-inflammatory, pro-angiogenic response. BSMC and EC may thus be promising new targets to counteract and/or alleviate airway wall remodeling.

## Introduction

Persistent airway wall remodeling is an important pathology of asthma, which is characterized by basement membrane thickening, increased bronchial smooth muscle mass and neovascularization. Together with enhanced extracellular matrix deposition these pathologies result in thickening of the basement membrane and the underlying tissue layers. It is of interest that BSMC from asthmatic patients have a distinct “phenotype” compared to cells from non-asthma subjects [1]. This disease specific phenomenon is associated with diminished expression of the CCAAT/enhancer binding protein (C/EBP)-α in BSMC of asthma patients [2]–[5]. Lower C/EBP-alpha levels may explain several parameters associated with the hyper-reactive (“primed”) phenotype of the asthmatic BSMC, including an increased release of pro-inflammatory factors and mediators [6], [7]. Smooth muscle changes in allergic asthma include myofibroblast differentiation, BSMC hyperplasia and hypertrophy and vascular smooth muscle thickening [8].

For oxygen supply, BSMC depend on direct diffusion though the basement membrane, as well as on oxygen delivered by micro-vessels present in the airway wall. In the asthmatic airway, local hypoxic conditions may occur due to increased thickness and altered composition of the basement membrane, as well as of edema, which may affect the rate of oxygen diffusion [9]. In healthy lungs, the thickness of the basement membrane measures 5–7 µm, while in asthmatics the size was found to be up to 5 times increased [10]. Even in young asthmatic children the basement membrane was significantly thicker compared to non-asthmatic controls [11]. The increased thickness of the basement membrane may readily induce a locally restricted hypoxic environment, which affects the properties and behavior of BSMC. In addition, increased oxygen consumption by inflammatory cells and growing BSMC may further add to locally restricted hypoxic conditions in the lung [9].

It has only recently been suggested that hypoxia may play a role in the pathogenesis of asthma. For instance, hypoxia aggravated airway inflammation, remodeling, and furthered the development of asthma in a murine model of asthma [12]. The activation of proteins of the hypoxia inducible factor (HIF)-family is a major regulator of the cellular response to hypoxia [13] and increased level of HIF-α, have been detected in endobronchial biopsies and in the broncho-alveolar lavage fluid (BALF) of asthmatic patients [12]. Similarly, increased levels of the HIF subunits (HIF-1α and HIF-1β) have been observed in lung tissues, epithelial cells and BALF of asthmatic patients and have been correlated with the level of VEGF [14].

In order to overcome hypoxia, resident cells of the airway wall, including BSMC and fibroblasts, may induce the production of angiogenic factors leading to the formation of new micro-vessels in the airway wall. VEGF, one of the most potent angiogenic factors, is indeed produced by smooth muscle cells [15]–[17] and has been shown to stimulate the proliferation of vascular smooth muscle cells [18], [19]. These findings indicate that BSMC are able to induce angiogenesis and may contribute to the observed neovascularization of the airway wall.

In our present study we examined the effects of hypoxia on inflammatory and proliferative responses of BSMC obtained from the lungs of both non-asthmatic and asthmatic subjects. Our data demonstrate that hypoxia diminished the proliferation-rate of BSMC, whereas it concomitantly induced HIF-1α and the subsequent release of VEGF, IL-6 and IL-8.

## Methods

### Ethics Statement

The use of human primary BSMC was approved by the local ethical committee (University Hospital, Basel, Switzerland). Written informed consent was provided by each patient. For the staining ethical approval for the clinical trial protocol and the use of human tissue was obtained. The study was approved by the Ethics Review Committee of the Central Sydney Area Health Service, Royal Prince Alfred Hospital zone (protocol number X02-0137).

### Patients

Patients with mild to moderate asthma (n = 7 total number participating in this study; 3 females/4 males; age 23–64 years) had reversible airway obstruction documented in the past, with a median FEV_1_ of 70.5% of the predicted value (ranging from 45.3% to 84.6%). The samples used as non-asthmatic controls (n = 6 total number participating in this study) were either from healthy organ donors whose lungs were deemed unfit for use in a transplant procedure or from the healthy part of lung resection material obtained after lung surgery.

### Cryosectioning, Staining and Light Microscopy of Endobronchial Biopsies

Superficial endobronchial biopsy specimens of the airway mucosa were immediately snap frozen in optimal cutting temperature (OCT) medium (Tissue-Tek, Sakura Finetechnical, Tokyo, Japan) on a cork disc by immersion in isopentane (2-methylbutane, HPLC grade, Sigma-Aldrich, Buchs, Switzerland) suspended in liquid nitrogen and kept at −80°C. Frozen biopsies were sectioned on a cryostat (Shandon Cryotome 620E) at −18°C into sections of 7 µm and stored at −80°C. The frozen sections were air dried at room temperature, fixed in 50% acetone/50% methanol (90 s) and stained with Harris’s haematoxylin and alcoholic eosin (Fronine Laboratory Supplies). Light microscopic photographic images were taken at 60× (Olympus AX70 microscope, DP50 camera).

### Isolation of Primary Bronchial Smooth Muscle Cells

BSMC were obtained from endobronchial biopsies obtained by flexible bronchoscopy or therapeutically resected lung tissue (Department of Internal Medicine, Pulmonology, University Hospital Basel, Basel, Switzerland). Isolation of BSMC was performed as described earlier [1], [20] with some modifications. BSMC bundles were dissected from the surrounding tissue using a binocular microscope. Muscle bundles were then placed in a 25 cm^2^-flask in 1 ml Dulbecco’s modified Eagle’s medium (DMEM) containing GlutaMax-I 4.5 g/l glucose (Gibco®, Bioconcept, Allschwil, Switzerland), 5% FCS, 1× antibiotics-antimycotics, and 1× modified Eagle’s medium vitamin mix (Invitrogen, Lubio, Luzern, Switzerland). BSMC were characterized by immunofluorescence as described earlier [21].

### BSMC Culture and Preparation of Conditioned Media

BSMC were grown in BSMC-growth medium (GM): RPMI 1640 supplemented with 5% fetal calf serum (FCS), 8 mM L-glutamine, 20 mM hydroxyethyl piperazine ethane sulfonic acid, 1× antiobiotics-antimycotics and 1× modified Eagle’s medium vitamin mix (Invitrogen, Lubio, Luzern, Switzerland) under normoxic conditions (21% O_2_, 5% CO_2_, 37°C) until they reached confluency and then subcultured. To study hypoxia, cells were cultured at oxygen concentrations of 1% and 5% (5% CO_2_, 37°C). For the preparation of conditioned medium (CM), BSMC were seeded at 10^5^ cells/well in 6-well plates and grown in normal growth medium for 24 h. Then, cells were subjected to a 24 h period of serum-deprivation and then further cultured 72 h under normoxic and hypoxic growth conditions. Cell counts were performed before stimulation and after 72 h. Culture supernatants (CM) were harvested, centrifuged to remove cells and stored at −20°C until use. For any given BSMC isolate the experimental protocols for preparation of CM were performed on at least two independent occasions and in duplicate for each condition. Normalized conditioned media (CM) used in endothelial cell sprout outgrowth assays was obtained from BSMC grown under hypoxic (1% O_2_, 5% CO_2_, 37°C, 72 hours) and normoxic conditions (21% O_2_, 5% CO_2_, 37°C, 72 hours). All experiments were performed at least in duplicates.

### Proliferation Experiments

To determine the effect of hypoxia on proliferation BSMC were seeded at 5×10^5^ cells/well in 12 well plates, allowed to adhere for 24 h in BSMC-GM, serum deprived for 24 h prior to stimulation and then cultured for 72 h in the presence or absence of 5% FCS under three different oxygen concentrations (21%, 5% or 1% O_2_). Cell counts were performed in duplicates before stimulation and after 72 h using a particle counter (Beckman Coulter particle counter Z1, Nyon, Switzerland).

### SDS-PAGE and Western Blot

Total protein lysates were size-fractioned on gradient (4–20%) Tris-HEPES gels (Pierce, Thermo-Scientific, Lausanne, Switzerland) and transferred to nitrocellulose membranes as described earlier [4]. Membranes were blocked for 30 min with 3% BSA in Tris buffered saline with 0.1% Tween 20 (TBST) for detection of PCNA, cyclin E, α-tubulin, GAPDH (Santa Cruz Biotech, Lucerna-Chem, Luzern, Switzerland) or for 1 h in 5% BSA in TBST for the detection of HIF-1α (Cell Signaling technologies, Bioconcept, Allschwil, Switzerland). Primary antibodies were applied for 2 h at RT in TBST/1%BSA (PCNA, cyclin E, α-tubulin, GAPDH) or overnight at 4°C in TBST/5%BSA (HIF-1α). HRP-conjugated species-specific secondary antibodies were applied for 30 min (PCNA, cyclin E, α-tubulin, GAPDH) in TBST or for 2 h in 3% milk powder in TBST (HIF-1α). Membranes were incubated with SuperSignal Western Pico Chemiluminescent Substrate (Pierce, Thermo-Scientific, Lausanne, Switzerland) for 5 min, the signal was detected on Fuijifilm Super RX X-ray films (Lucerna-Chem, Luzern, Switzerland), and developed in a Curix60 film-processor (Agfa, Dübendorf, Switzerland).

### Exclusion of Apoptosis

Western-analysis was performed to detect caspase-3 (p32 and p17) (H-277; Santa Cruz Biotech, Lucerna-Chem, Luzern, Switzerland). The ratio p32:p17 was used as an index of apoptosis: A decreased ratio is indicative of apoptosis. Using this method we observed that hypoxia (1% O2, 48 h) did not affect apoptosis.

### Cytokine-ELISA

Enzyme linked immunosorbent assay (ELISA) kits for ENA-78, IL-6, IL-8 and VEGF-A were purchased from R&D Systems (Abingdon, UK) and performed according to the manufactureŕs instructions. Cytokine levels were determined in 100 µl of CM obtained from BSMC and were measured undiluted (VEGF-A), 1∶5 diluted (ENA-78, IL-6) or 1∶50 diluted (IL-8). Cytokine concentrations were normalized for cell numbers and expressed as per 10^5^ cells.

### Endothelial Tube-formation Assay

Human microvascular endothelial cells (EC) HMEC-1 [22] were normally maintained in EC growth medium (ECGM, Provitro, Bioconcept, Allschwil, Switzerland) supplemented with 10% FCS under normoxic conditions. Spheroids composed of 500 HMEC-1 cells were prepared using the hanging drop method [23]. The tube-forming (sprout outgrowth) assay was performed as previously described [24]. At least 10 spheroids per gel were embedded within fibrin gels in 48-well plates. Gels were overlaid with a 1∶1 mixture of ECGM supplemented with 2% FCS and either BSMC medium (to determine spontaneous sprout outgrowth) or CM obtained from BSMC grown under hypoxic (1% O_2_) and normoxic (21% O_2_) conditions. In experiments studying the effects of the CXCR2 antagonist SB 265610 (Sigma-Aldrich, Buchs, Switzerland) the inhibitor was added to the gel 1 h prior to stimulation with CM. VEGF-A neutralizing antibody (R&D, Abingdon, UK) was added to CM for 1 h (37°C) prior to its addition to the gels containing the spheroids. After incubation for 24 h, spheroids were fixed in-gel, stained with TRITC-conjugated phalloidin (Sigma-Aldrich, Buchs, Switzerland) and sprout outgrowth from each spheroid was quantitated by morphometric analysis of the length of outgrowing tubules [24]. The length of the 10 longest tubules per spheroid of 7 randomly chosen spheroids were quantitated by morphometric analysis using AnalySIS software (Soft Imaging System GmbH, Munich, Germany) and the mean ± S.E.M. was calculated.

### Statistics

Cytokine and proliferation data are presented as mean ± S.E.M, Western blot analysis is shown as mean ± S.E.M after densitometric image analysis (ImageJ software, National Institute of Mental Health, Bethesda, Maryland, USA). Assuming normal distribution of our datasets, paired or unpaired, two-tailed student’s t-test was performed and p-values <0.05 were considered significant.

## Results

### Increased Basement Membrane Thickness in Human Tissue Sections from the Lung of Asthma Patients

**Figure 1:**
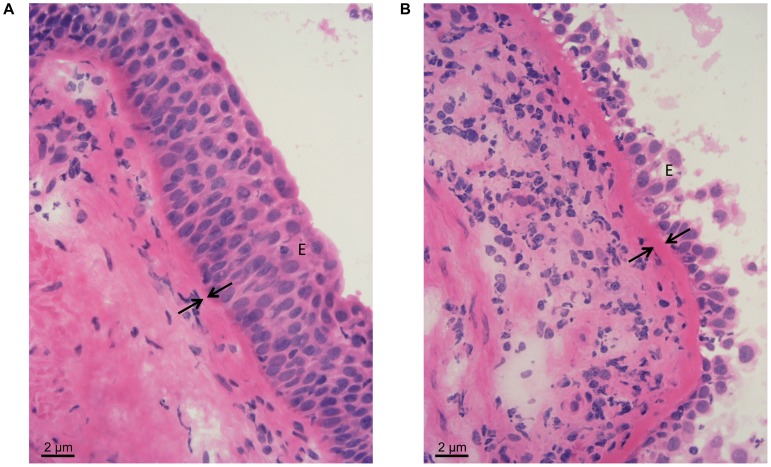
Light microscopic photographic images of airway tissue sections obtained from a non-asthmatic (A) and an asthmatic (B) patient. Images (magnification 60×) are representative of tissues obtained from 3 non-asthmatic and 7 asthmatic patients stained with Haematoxylin-Eosin. Note the increased thickening of the basement membrane in the asthmatic airways. E = epithelium, arrows are indicating the basement membrane.

Superficial endobronchial biopsy specimens were stained using Harris’s haematoxylin and alcoholic eosin and light microscopic photographic images were recorded. Figure 1 presents a typical example of a human airway tissue cross-section demonstrating the increased thickness of the basement membrane present in non-asthma (Figure 1A) relative to asthma (Figure 1B) subjects.

### Hypoxia Reduced Proliferation of BSMC of both Asthmatic and Non-asthmatic Subjects

**Figure 2:**
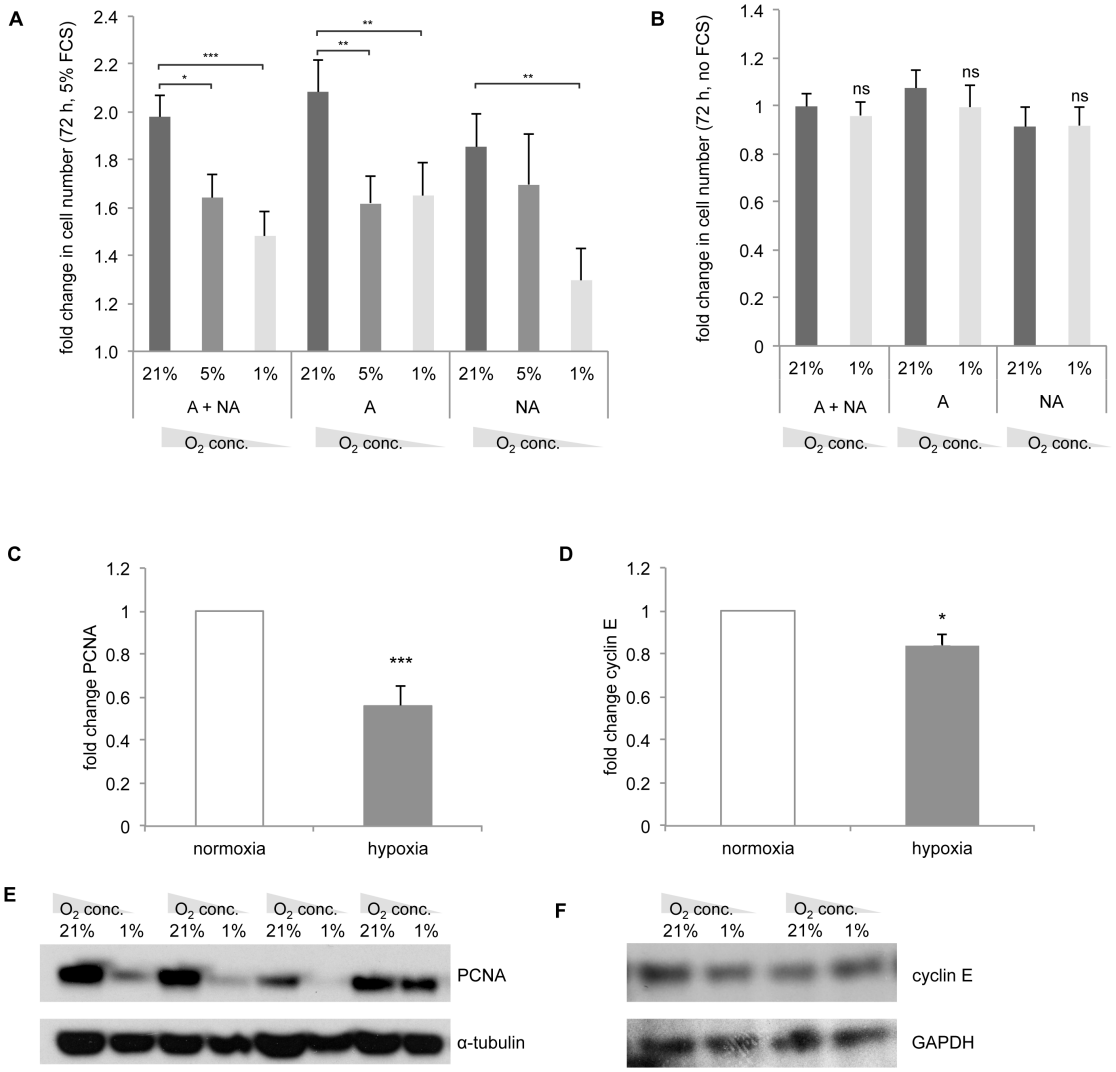
Proliferation characteristics of BSMC from asthmatic (A) and non-asthmatic (NA) subjects in the presence of 1%, 5% and 21% O_2_ (72 h). BSMC were cultured for 72 hours in presence (**A**) and absence (**B**) of 5% FCS. The data presented in **A** and **B** are shown as one group (6A+6NA for 21% O2 and 4A+3NA for 1% and 5% O_2_), as well as two separate groups (6A and 6NA for 21% O_2;_ 4A and 3NA for 1% and 5% O2). **C** and **D,** densitometric analysis of PCNA (9 independent experiments in 5 subjects) and cyclin E (6 independent experiments in 5 subjects) under hypoxic (1% O_2_) and normoxic conditions (21% O_2_). **E** and **F,** representative Western blots for PCNA and cyclin E protein expression. Densitometric values are given as mean ± SEM (*p-value ≤0.05, **p-value ≤0.01, ***p-value ≤0.001).

BSMC obtained from asthmatic (n = 4) and non-asthmatic (n = 5) subjects were cultured for 72 hours under either normoxic (21% O_2_) or hypoxic conditions (5% and 1% O_2_) in presence and absence of 5% FCS. As shown in Figure 2A, in the presence of 5% FCS and 21% oxygen (i.e. normoxic conditions) BSMC numbers significantly increased in both asthmatics (2.08±0.13-fold) and non-asthmatics (1.85±0.13-fold). This FCS-induced proliferation was significantly reduced in the presence of 5% O_2_ in BSMC of asthmatics (1.62±0.12-fold (p<0.05), but not in BSMC of non-asthmatics. Severe hypoxia (1% O_2_) did not further reduce BSMC proliferation of asthmatics (1.65±0.14-fold), but now a similar significant reduction of proliferation was observed in BSMC of non-asthmatics (1.30±0.13-fold; p<0.05). Without FCS, BSMC did not proliferate and therefore hypoxia (1% O_2_) did not have any effect (figure 2B). As shown in figure 2C- 2F, in the presence of 5% FCS and 21% oxygen (i.e. normoxic conditions) the proliferation markers PCNA and cyclin E were both significantly down regulated (PCNA: 56±1% compared to 21% O_2_, p<0.001 and cyclin E: 84±5% compared to 21% O_2_, p<0.05). No differences were observed between the asthmatic and non-asthmatic subjects.

### Hypoxia Induced HIF-1α in BSMC

**Figure 3:**
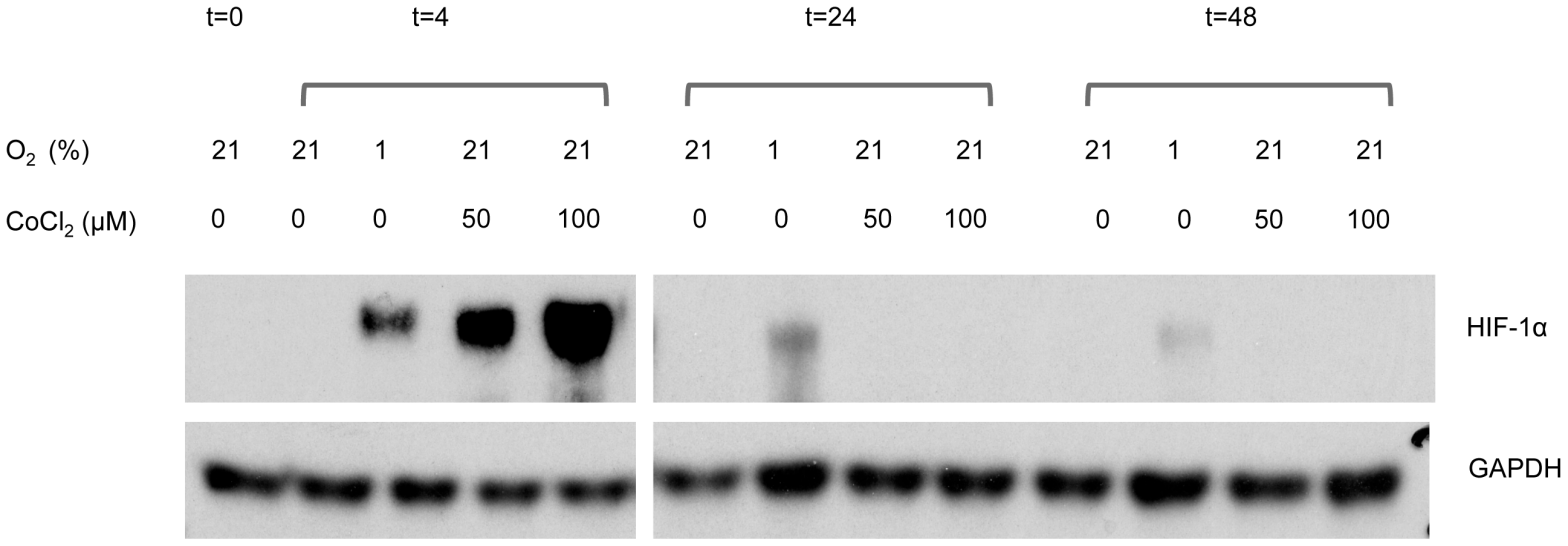
Western blot analysis detecting HIF-1α and GAPDH (loading control) in lysates of BSMC. Cells were incubated under 1% and 21% O_2_ for 4, 24 and 48 h (indicated at top). CoCl_2_ was used as positive control to stabilize HIF-1α protein. In contrast to CoCl_2_, which has a transient effect only, hypoxia induced a prolonged expression of HIF-1α. The experiments shown are representative for 5 independent experiments. The right hand panel (showing the 24 h and 48 h expression levels of HIF-1α) was obtained after longer exposure of the same blot, which contained all samples.

To study whether hypoxia was associated with the induction of HIF-1α, BSMC (n = 5) were cultured in the presence of 5% FCS and under normoxic (21% O_2_), or stringent hypoxic conditions (1% O_2_). As demonstrated in figure 3, normoxic conditions did not induce HIF-1α. In contrast, HIF-1α was transiently induced and peaked after 4 h in BSMC under stringent hypoxic conditions. Extended exposure to hypoxic conditions (24 and 48 h) HIF-1α levels decreased, but did not return to control levels. Cobalt chloride, a chemical inhibitor of the prolyl-hydroxylase that targets HIF-1α for proteasomal degradation under normoxic conditions, was used as a positive control (50 and 100 µM).

### Hypoxia Induced IL-6, IL-8 and VEGF-A, but not ENA-78

**Figure 4:**
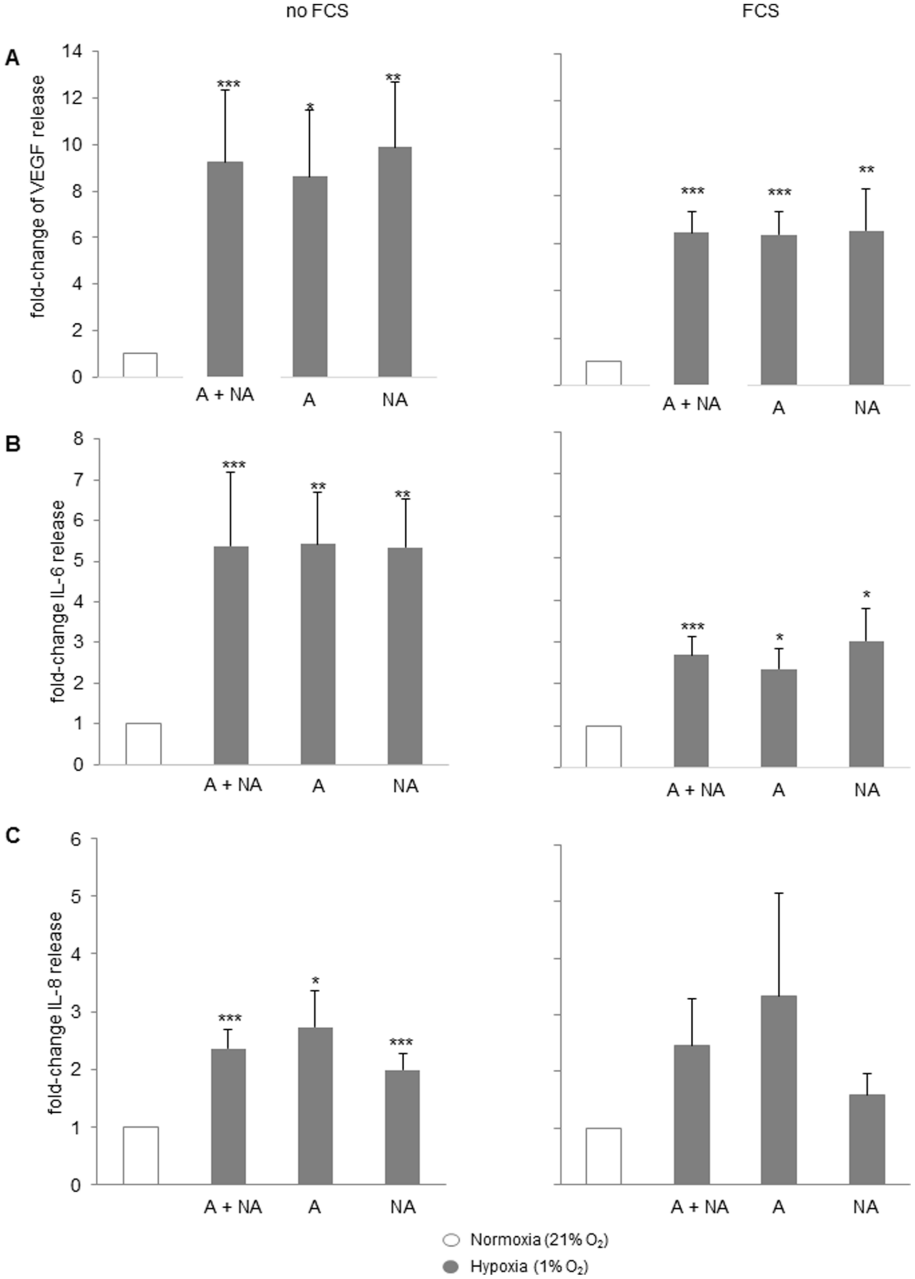
Normoxia (21% O2) versus hypoxia (1% O2). Effects on cytokine release from BSMC grown in the presence of 5%FCS (72 h). Concentrations of VEGF (**A**), IL-6 (**B**) and IL-8 (**C**) in CM collected from BSMC of 5 non-asthmatic and 5 asthmatic subjects were determined by ELISA. Values are given as mean ± SEM. *p-value ≤0.05, *p-value ≤0.01, ***p-value ≤0.001 (all relative to 21% O_2_). Abbreviations: A = asthmatics, NA = non-asthmatics.”

Next, we studied the effect of hypoxia on the release of four angiogenic/pro-inflammatory cytokines: IL-6, IL-8, VEGF-A and ENA-78. BSMC obtained from asthmatic (n = 5) and non-asthmatic (n = 5) subjects were cultured for 72 h under either normoxic (21% O_2_) or stringent hypoxic conditions (1% O_2_) in presence or absence of 5% FCS. In the absence of FCS, hypoxia significantly increased the release of VEGF-A protein in both asthmatic and non-asthmatic cells (8.62±2.86-fold and 9.88±2.79-fold; both p<0.05; Figure 4A). Similarly, under the same conditions the release of IL-6 protein increased in both asthmatic and non-asthmatic cells (5.42±1.29-fold and 5.34±1.18-fold, respectively; both p<0.05; Figure 4B). In the absence of FCS, hypoxia did not affect the release of IL-8 (Figure 4C).

In the presence of 5% FCS, hypoxia significantly increased the release of VEGF-A protein by both asthmatic and non-asthmatic cells (6.37±0.96-fold and 6.54±1.74-fold; both p<0.05; figure 4A). Under the same conditions the release of IL-6 protein increased in both asthmatic and non-asthmatic cells (2.36±0.59-fold and 3.03±0.76-fold; both p<0.05; figure 4B). The effect of hypoxia on the release of IL-8 was less pronounced, but significant (2.72±0.63-fold and 1.98±0.29-fold; both p<0.05; Figure 4C). Hypoxia did not affect the release of ENA-78 significantly (0.88±0.24-fold, p = 0.59). No significant differences were observed between asthmatic and non-asthmatic BSMC in any condition.

### Increased Angiogenic Potential of BSMC Grown under Hypoxic Conditions

**Figure 5:**
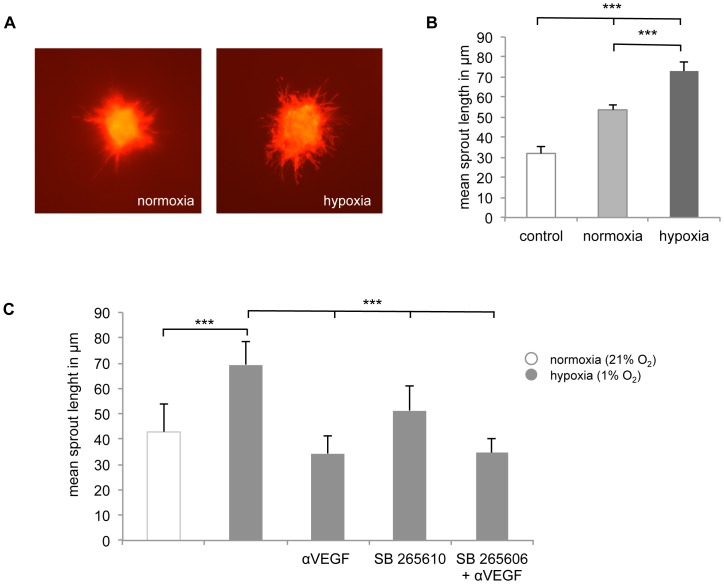
*In vitro* angiogenesis assay. **A,** Representative images of the *in vitro* angiogenesis assay showing sprout outgrowth from EC-spheroids incubated with CM from BSMC cultured in presence of 21% O_2_ (normoxia) or 1% O_2_ (hypoxia). **B,** lengths of sprouts outgrowing from every spheroid were measured and the mean of the longest 10 sprouts of 7 randomly chosen spheroids was plotted. **C,** effects of the CXCR2-blocker SB 265610, anti-VEGF, and the combination of SB 265610 plus anti-VEGF on EC-sprouting using the *in vitro* angiogenesis assay. EC-sprouting was induced by pooled CM of BSMC (n = 5) cultured under hypoxic conditions (1% O_2_, 72 h). Values are given as mean of the mean ± SEM (*p-value ≤0.05, **p-value ≤0.01, *** p-value ≤0.001).

The angiogenic potential of CM collected from BSMC grown under hypoxic (1% O_2_) and normoxic (21% O_2_) conditions were examined using the EC-spheroid *in vitro* angiogenesis assay. Spheroids were also cultured in unconditioned medium (i.e. with medium that had not been included with BSMC) to control for “spontaneous” sprout outgrowth. Figure 5 shows representative images of spheroids incubated with CM from BSMC grown under normoxic (left panel) or hypoxic (right panel) conditions. The quantitation of sprout outgrowth as the mean tubule length/spheroid after a 24 h culture period is shown in Figure 5B. CM of BSMC cultured under hypoxic conditions significantly increased the sprout formation compared to CM of BSMC grown under normoxic conditions (53.7±2.4 µm *vs.* 73.0±4.5 µm; p<0.001; n = 3).

### Hypoxia-induced Angiogenesis is Reversed by SB265610 and anti-VEGF

Finally, we sought to define the mechanism involved in the aforementioned findings. Since IL-8 signals through the chemokine receptor CXCR2, we studied the effect of a competitive CXCR2-selective antagonist SB 265610 (10 nM) and a neutralizing anti-VEGF antibody (0.2 µg/ml) in the EC-spheroid *in vitro* angiogenesis assay. We examined the effect of hypoxia on pooled CM, derived from asthmatics and non-asthma controls. CM from non-asthma controls did not show any difference whether BSMC were grown under hypoxic or normoxic conditions. In contrast, CM from asthmatics significantly increased the sprout outgrowth from EC spheroids comparing normoxia (42.83±11.06 µm) and hypoxia (69.39±9.22 µm; p<0.001). As demonstrated in figure 5C, SB 265610 and anti-VEGF significantly reduced CM-induced EC-sprouting (SB 265610: 51.31±9.77 µm, p<0.05; anti-VEGF 34.33±7.09 µm, p<0.001).

## Discussion

Hypoxia of the airways can be caused by a range of mechanisms, including diminished ventilation drive, airway obstruction, intra-alveolar exudates, airway wall inflammation, as well as fibrosis and basement membrane thickening [25]. The latter is one of the striking features of the asthmatic airway wall and may well contribute to locally restricted hypoxia. This may in turn affect the properties of resident cells present in the airway wall. Biopsy studies in adults and children showed a significant increase in the number of micro-vessels present in the airway wall of asthma patients and there is evidence that endothelial cells undergo proliferation [26]–[28]. In our current study, we showed that hypoxia has a profound effect on the proliferation of and angiogenic factors released by primary human BSMC of both asthmatic and non-asthmatic subjects.

First, hypoxia has the capacity to significantly reduce the proliferation rate of BSMC of both asthmatic and non-asthmatic subjects. Importantly, in the presence of 5% O2 the proliferation of BSMC of asthma patients was significantly reduced, whereas the same conditions did not affect BSMC of control subjects. Stringent hypoxic conditions (1% O_2_) significantly reduced the proliferation in cells of both asthmatics and non-asthmatics. This indicates that BSMC of asthmatics are more responsive to hypoxic environments compared to non-asthmatics. The reason for this difference is unclear, but it might reflect diminished proliferation control responses as observed in cultured asthmatic BSMC [2]–[5], [29]. The reduced proliferation under stringent hypoxic conditions (1% O_2_) was confirmed by lower levels of PCNA and cyclin E proteins in BSMC of both asthmatic and non-asthmatic subjects. Our data also indicate that the effects of hypoxia may be distinct in different model organisms, since it has been demonstrated that BSMC of rats have significantly increased proliferation rates under hypoxic conditions [30]. The reason for this discrepancy between primary human BSMC and those of rats is currently not known.

Second, our study showed that hypoxia enhanced the release of VEGF, a mediator of micro-vascular leakage, EC proliferation and vascular remodeling, as well as IL-6 and IL-8, which are known to be involved in angiogenesis [31]–[33]. The mechanisms underlying airway wall remodeling present in the asthmatic lungs, which also involve angiogenesis of the airway wall, are complex and incompletely understood. Hypoxia may trigger the BSMC to express HIF-α, a transcription factor critically involved in the regulation of several inflammatory and pro-angiogenic factors. Both VEGF and HIF-α are expressed in the airways and airway lining fluids of asthma patients, suggesting that both factors may play an important role in both inflammation and vascular remodeling in asthma [34], [35]. The observed increased number of micro-vessels present in the airway wall of patients with asthma [26]–[28], may be explained by the significant higher levels of VEGF and increased HIF expression in the sub-epithelial cell layers of asthma patients relative to healthy controls [14]. It is important to note that high expression of VEGF was reported in the airways of asthma patients [35], [36].

Our results demonstrated that hypoxia transiently induced HIF-1α protein expression in BSMC, with no differences between cells of asthmatic and non-asthmatic subjects. The expression of HIF-1α, the hypoxia-induced subunit of the hypoxia inducible factor (HIF), was transient and optimal after 4 hours, indicating that the effects on the release of VEGF, IL-6 and IL-8 might be via this transcription factor. CM isolated from BSMC cultured under hypoxic conditions demonstrated a significantly increased endothelial sprout outgrowth relative to CM of BSMC grown under normoxic conditions, confirming that BSMC cultured under hypoxic conditions induced the release of pro-angiogenic factors. Indeed, our blocking experiments using SB265610 and/or anti-VEGF demonstrated both the involvement of CXCR2 signaling and VEGF in the angiogenic process.

Previously, we showed that BSMC isolated from asthma patients exhibit increased angiogenic potential, which associated with increased productions of CXCR2 ligands (ENA78, GRO-α and IL-8) and VEGF [37]. Our present study showed that hypoxia predominantly induced VEGF and IL-6, whereas IL-8 was only 2-fold increased and ENA-78 not affected. It has been observed that hypoxia regulates VEGF activities mainly through transcriptional repression of the neuropilin-2 receptor [38]. Furthermore, increased levels of IL-6 and IL-8 have been reported in asthma patients [39]. The involvement of HIF-1α in the increased release of IL-6 and IL-8 is largely unknown, although both genes are associated with hypoxia-inducible promoter and/or enhancer sequences [own observations after checking the annotated sequences with GENE tools; IL6 annotation: Chromosome 7, NC_000007.13 (22766766.22771621); IL8 annotation: Chromosome 4, NC_000004.11 (74606223.74609433)]. This suggests that hypoxia may exert differential effects on different cytokines via distinctly different mechanisms.

In conclusion, hypoxia has dualistic effect on proliferative and inflammatory responses of both non-asthmatic and asthmatic primary human BSMC. Our data imply that hypoxia cannot be a direct cause for the observed increased smooth muscle mass in the airway wall of asthma patients. Rather, these cells start to elicit a range of pro-inflammatory cytokines and growth factors that intensify airway wall inflammation and remodeling through advancing the process of neovascularization. A novel strategy to counteract airway wall remodeling may therefore be found in the development of drugs that counteract the process of neovascularization. Both BSMC and EC are therefore promising new targets to counteract and/or alleviate airway wall remodeling.
